# The mediating effect of job burnout between workplace violence and sleep disorders among emergency department nurses

**DOI:** 10.3389/fpubh.2025.1640865

**Published:** 2025-09-01

**Authors:** Jizhou Mao, Zhihan Gu, Luying Zhong, Hao Zhang, Xiaoli Chen, Liqun Zou

**Affiliations:** ^1^Department of Equipment, West China Hospital, Sichuan University, Chengdu, China; ^2^Department of Emergency Medicine, West China Hospital, Sichuan University, Chengdu, China; ^3^West China School of Nursing, Sichuan University, Chengdu, China; ^4^Disaster Medical Center, Sichuan University, Chengdu, China

**Keywords:** emergency department nurse, job burnout, workplace violence, sleep disorder, mediating effect

## Abstract

**Background:**

Sleep disorders severely impact the health of emergency department nurses and the quality of nursing care. Workplace violence is a significant contributing factor to the occurrence of sleep disorders among nurses.

**Objective:**

To investigate whether job burnout plays a mediating role between workplace violence and sleep disorders among emergency nurses, and to determine the degree of interaction between workplace violence, job burnout and sleep disorders.

**Method:**

A cross-sectional survey study was conducted from December 2023 to January 2024, in which 1,540 emergency department nurses in China were surveyed using workplace violence assessment, job burnout assessment and sleep disorder assessment questionnaires. Spearman correlation analysis was employed to assess the relationships among workplace violence, job burnout, and sleep disorders. Mediation structural equation modeling analysis was performed using the PROCESS macro in SPSS which is a plug-in special for mediating and moderating effect analysis.

**Results:**

Among the 1,540 emergency department nurses surveyed, 59.3% experienced sleep disorders. Workplace violence showed a positive correlation with job burnout (*r* = 0.529, *p* < 0.01) and with sleep disorders (*r* = 0.950, *p* < 0.01). Job burnout was also positively correlated with sleep disorders (*r* = 0.343, *p* < 0.01). Workplace violence significantly predicted job burnout (*β* = 1.658, *p* < 0.01) and sleep disorders (*β* = 0.250, *p* < 0.01). Job burnout significantly predicted sleep disorders (*β* = 0.132, *p* < 0.01). The overall mediating effect of job burnout between workplace violence and sleep disorders accounted for 46.7% of the total effect, and this mediating effect was statistically significant.

**Conclusion:**

Job burnout has mediated the impact of workplace violence on sleep disorders among emergency nurses. This indicates that intervening in job burnout among emergency department nurses is a key pathway to reducing the occurrence of their sleep disorders. Simultaneously, combining interventions targeting workplace violence may yield more comprehensive and efficient results. Future research should employ longitudinal designs to examine the impact trajectories of workplace violence on job burnout and sleep disorders among emergency nurses.

## 1 Introduction

Shift work often leads to circadian misalignment, resulting in varying degrees of sleep issues among nurses (1). A study indicated that about 39.2% of healthcare workers experience sleep disorders, surpassing the global prevalence of 27% as reported by the WHO (2, 3). Emergency department nurses, facing intense work pressure, extended night shifts, and frequent rotations, exhibit notably poorer sleep quality (4). Research by Stimpfel et al. (5) revealed significant links between sleep status and nursing quality, with poor sleep quality correlating with low mood, heightened stress levels (6), and adverse effects on both nurses’ health and job performance, subsequently impacting patient care (5, 7). Sleep disorders are associated with psychiatric, metabolic, and cardiovascular conditions (8, 9), posing a higher risk for metabolic disorders among the predominantly female nursing workforce (10). Identifying the mechanisms behind sleep disorders is crucial for enhancing emergency medical services and safeguarding the well-being of emergency department nurses.

Workplace violence (WPV) significantly contributes to the onset of sleep disorders. A considerable number of nurses encounter workplace incivility, lateral violence, and various forms of WPV (11). Approximately 10% of nurses who are victims of bullying develop symptoms of post-traumatic stress disorder (PTSD) (12). Moreover, nurses exposed to frequent violence are at an increased risk of developing sleep disorders (13). Liu Fang et al. established a correlation between stress and sleep using the preventive stress management model (14). Studies also point out that workplace violence plays a crucial role in job burnout in the United States (15). Nurses who face higher levels of physical and verbal aggression tend to experience elevated levels of job burnout (16). A study conducted in China demonstrates that workplace violence impacts clinical nurses’ job burnout through both independent and sequential mediating effects of intrusive thoughts and ego depletion (17). Furthermore, evidence-based medicine demonstrates that higher levels of nurse burnout are associated with a greater prevalence of sleep disorders (18). Burnout compels nurses to utilize longer breaks and leisure time to recover lost energy (19), leading to alterations in sleep architecture and reductions in both the quality and quantity of sleep (20). Due to factors such as patients awaiting urgent treatment, acute psychiatric episodes, and alcohol intoxication, emergency departments are high-incidence settings for workplace violence (21). Therefore, investigating the mechanisms linking workplace violence to sleep disorders among emergency department nurses is imperative.

A review of existing domestic and international studies indicates that workplace violence’s impact on the sleep patterns of emergency nurses may be influenced by intermediary factors rather than being purely direct. There are observed correlations among workplace violence, nurses’ sleep quality, and job burnout. However, the causal pathways linking workplace violence, burnout, and sleep disturbances among emergency department nurses have not been extensively investigated. It remains unclear whether job burnout can act as a mediator between workplace violence and the sleep quality of emergency nurses. Therefore, based on the Preventive Stress Management model (22), this study proposes a framework in which the work environment (represented by workplace violence) functions as the independent variable, the health outcome (sleep disorders) as the dependent variable, and individuals’ immediate reaction (job burnout) as the mediating variable. The Preventive Stress Management model points out that the pressure process is from the pressure source to the pressure reaction and then to the health. Stressors are causal factors which may be environmental or self-imposed. Exposure to a pressure source can cause a pressure response; the stress response may be positive, leading to benign stress, or it may be negative, leading to adverse outcomes. We hypothesize that: (I) Workplace violence, job burnout, and sleep disorders are interrelated; (II) Job burnout acts as a mediator in the relationship between workplace violence and sleep disorders among emergency nurses. This study aims to validate hypotheses to explore the pathway mechanisms through which workplace violence affects sleep disorders among emergency department nurses, determining the mediating role of job burnout, thereby providing a theoretical foundation for the development and implementation of relevant intervention measures.

## 2 Methods

This study constitutes a secondary analysis of cross-sectional survey data originally collected for an investigation on the health and sleep status of emergency nurses and their related influencing factors. Utilizing this dataset, our research team has to date published four peer-reviewed articles. These publications respectively: gender differences in work–family conflict among emergency nurses (23); the impact of workplace violence on nurse health outcomes (24); the mediating role of work–family conflict between occupational stress and health in emergency nurses (25); the effects of effort-reward imbalance on the health of emergency department nurses (26).

### 2.1 Study participants

A stratified cluster sampling method was used to recruit emergency department nurses based on China ‘s geographical regions (Northeast, East China, North China, Central China, South China, Southwest and Northwest) between December 2023 and January 2024. Inclusion criteria were: ① Registered nurses working in emergency departments; ② ≥1 year of emergency nursing experience. Exclusion criteria were as follows: ① History of psychiatric disorders; ② Nurses in training programs; ③ Nurses on extended medical/maternity leave or breastfeeding (≥1 month). Based on preliminary survey results and assuming a significance level (α) of 0.05 with 80% statistical power, the required sample size was calculated as 1,330 participants.

### 2.2 Research tools

#### 2.2.1 Demographic questionnaire

A self-designed demographic questionnaire developed by the research team was administered, collecting data on gender, age, education level, marital status, childbearing status, smoking habits, alcohol consumption habits, work years in emergency nursing, professional title, weekly working hours, shift schedule patterns, and monthly income.

#### 2.2.2 Workplace violence assessment

Workplace violence was assessed using the Workplace Violence Questionnaire adapted by Hesketh et al. (27). This instrument contains 4 items: physical violence from patients or their families, psychological violence from patients or their families, physical violence from supervisors or colleagues, psychological violence from supervisors or colleagues. Each item employs a 7-point Likert scale (0–6). Respondents reported the frequency of workplace violence experienced during the past year. Higher scores indicate greater frequency of workplace violence exposure among emergency nurses. The questionnaire demonstrated acceptable internal consistency with a Cronbach’s α coefficient of 0.71.

#### 2.2.3 Job burnout assessment

The assessment of burnout among emergency nurses was conducted using the Chinese version of the Maslach Burnout Inventory-General Survey (MBI-GS) revised by Zhang et al. (28). This scale comprises three dimensions: emotional exhaustion, cynicism, and professional efficacy, with a total of 16 items, responses are recorded on a 7-point Likert scale (0–6). The emotional exhaustion and cynicism dimensions use forward scoring, while the professional efficacy dimension uses reverse scoring. The composite burnout score is calculated as [0.4 × emotional exhaustion score + 0.3 × cynicism score + 0.3 × (6 – professional efficacy score)]. A total burnout score < 1.5 is classified as no burnout, a score of 1.5–3.5 is classified as suspected burnout, and a score ≥ 3.5 is classified as burnout. The Cronbach’s α coefficients for the three dimensions and the questionnaire were 0.914, 0.814, 0.899, and 0.860, respectively. The results of the scale reliability analysis of the data in this study are as follows. The Cronbach’s α coefficient of the scale is 0.904, three dimensions are 0.964, 0.967, and 0.960, respectively.

#### 2.2.4 Sleep disorder assessment

Sleep status was evaluated using the Self-administered Sleep Questionnaire (SSQ) (29). This instrument assesses three sleep domains through specific items: sleep latency (time to fall asleep), sleep maintenance (continuity throughout the night) and early morning awakening. Each item is scored on a 5-point Likert scale (0–4). Participants were classified as having sleep disorders if they met ≥1 of the following criteria: taking >30 min to fall asleep, experiencing sleep maintenance problems “nearly every night” and waking up too early in the morning. Higher total scores indicate greater severity of sleep disorders. The scale demonstrated good reliability with a Cronbach’s α coefficient of 0.786. The Cronbach’s α coefficient of the data in this study is 0.809.

### 2.3 Data collection

Electronic questionnaires were created using Wenjuanxing (an online survey platform), which included an informed consent form. After obtaining approval from the participating hospitals and departments, the head nurses distributed the electronic survey link to emergency department nurses at their respective hospitals, having first explained the study’s purpose. Participation was voluntary. Only one submission per IP address was permitted, and all questions required completion before submission. After collection, invalid questionnaires with identical responses across all items were excluded.

### 2.4 Statistical analysis

Statistical analysis of the survey data was performed using IBM SPSS Statistics 26.0 software. Continuous variables are presented as mean ± standard deviation (*x-* ± *s*), and categorical variables are described using frequency counts and percentages (*n*, %). Pearson correlation analysis was employed to examine the relationships and directions of association among workplace violence, job burnout, and sleep disorder. The PROCESS macro was utilized to explore the mediating effect of job burnout on the relationship between workplace violence and sleep disorders. Firstly, the study estimated the direct effect of path a workplace violence (predictor) on job burnout (mediator). Secondly, this study estimated the direct effect of path b job burnout (mediating variable) on sleep disorders (outcome variable). Third, the product of the quantization path a and b (ab) is studied to obtain the indirect effect. The Bootstrap method (with 5,000 resamples) was applied to test the significance of the mediating effect, with the significance level set at *α* = 0.05 (two-tailed).

### 2.5 Ethical considerations

This study obtained ethical approval from the Ethics Review Committee of West China Hospital, Sichuan University [Approval No.: 2024 Audit (309)]. All participants provided informed consent and voluntarily participated in the research. This questionnaire does not collect any personally identifiable information, and only statistical summary numbers appear in the research report.

## 3 Results

### 3.1 Basic characteristics of the study participants

A total of 1,540 emergency department nurses from 30 hospitals were ultimately included in the data analysis. The 30 hospitals were distributed as follows: four hospitals each in North China, Central China, Northeast China, Southwest China, and Northwest China, five hospitals each in Southern China and Eastern China. Among the 1,540 emergency department nurses, 1,211 were female nurses (78.6%), and 1,316 were under 40 years old (85.5%). Junior-level titles accounted for 58.9% of the emergency department nurses. Details are presented in Table 1.

**Table 1:** Basic characteristics of the study participants (*n* = 1,540).

Variable	Category	*N*	Percentage (%)
Age	20–29 years	582	37.8
	30–39 years	734	47.7
	40–49 years	183	11.9
	≥50 years	41	2.7
Gender	Female	1,211	78.6
	Male	329	21.4
Marital status	Unmarried	560	36.4
	Married	980	63.6
Educational level	Associate degree	186	12.1
	Bachelor’s degree or above	1,354	87.9
Professional title	Junior	907	58.9
	Middle	574	37.3
	Senior	59	3.8
Years of nursing experience	≤5 years	491	31.9
	6–10 years	493	32.0
	11–15 years	300	19.5
	16–20 years	121	7.8
	>20 years	135	8.8
Working hours per week	<40 h per week	577	37.5
	41-48 h per week	780	50.6
	49-58 h per week	123	8.0
	≥59 h per week	60	3.9
Number of night shift	0 times per month	181	11.8
	1–4 times per month	239	15.5
	5–8 times per month	659	42.8
	≥9 times per month	461	29.9
Monthly income	<4,000 RMB per month	57	3.7
	4,000–5,999 RMB per month	149	9.7
	6,000–7,999 RMB per month	250	16.2
	8,000–9,999 RMB per month	368	23.9
	≥10,000 RMB per month	716	46.5
Workplace violence	Yes	1,309	85.0
	No	231	15.0
Job burnout	Yes	882	57.3
	Suspected	327	21.2
	No	331	21.5
Sleep disorders	Yes	913	59.3
	No	627	40.7

### 3.2 Scores of the measurement scales

The workplace violence score among emergency nurses was (2.2 ± 1.97). Notably, 61.3% of emergency nurses reported experiencing workplace violence within the past year. The composite job burnout score was (4.77 ± 6.16), with job burnout detected in 57.3% of the emergency nurses. The sleep disorder score was (8.56 ± 3.12), indicating that 59.3% of the emergency nurses experienced sleep disorders. The scores for each dimension of the workplace violence and job burnout scales are presented in Table 2.

**Table 2:** Scores of the measurement scales.

Variables	Min	Max	Scores $\bar{x}\pm s$
Workplace violence	0	12	2.2 ± 1.97
Physical violence	0	6	0.76 ± 0.98
Psychological violence	0	6	1.44 ± 1.17
Job burnout score	−9	22.8	4.77 ± 6.16
Emotional exhaustion	0	30	11.3 ± 7.76
Cynicism	0	30	8.61 ± 7.58
Professional efficacy	0	36	13.79 ± 10.04
Sleep disorder	3	15	8.56 ± 3.12
Sleep latency	1	5	2.85 ± 1.13
Sleep maintenance	1	5	2.83 ± 1.24
Early morning awakening	1	5	2.88 ± 1.30

### 3.3 Correlation analysis among workplace violence, job burnout, and sleep disorders

Correlation analysis revealed that workplace violence, job burnout, and sleep disorders among emergency department nurses were significantly pairwise correlated (all *p* < 0.01). Workplace violence was positively correlated with job burnout (*r* = 0.529, *p* < 0.01). Workplace violence was positively correlated with sleep disorders (*r* = 0.295, *p* < 0.01). Job burnout was positively correlated with sleep disorders (*r* = 0.343, *p* < 0.01). Table 3 presents the results of the correlation analyses for each dimension of workplace violence, job burnout, and sleep disorders.

**Table 3:** Correlation analysis among workplace violence, job burnout, and sleep disorders.

Variables	1	2	3	4	5	6	7	8	9	10	11
Physical violence	1										
Psychological violence	0.674^**^	1									
Workplace violence	0.899^**^	0.929^**^	1								
Emotional exhaustion	0.426^**^	0.624^**^	0.583^**^	1							
Cynicism	0.476^**^	0.606^**^	0.597^**^	0.878^**^	1						
Professional efficacy	0.023	−0.073^**^	−0.031	−0.096^**^	0.013	1					
Job burnout score	0.379^**^	0.574^**^	0.529^**^	0.876^**^	0.806^**^	−0.533^**^	1				
Sleep latency	0.138^**^	0.161^**^	0.164^**^	0.207^**^	0.207^**^	0.043	0.160^**^	1			
Sleep maintenance	0.230^**^	0.318^**^	0.303^**^	0.408^**^	0.391^**^	−0.005	0.352^**^	0.489^**^	1		
Early morning awakening	0.208^**^	0.295^**^	0.279^**^	0.401^**^	0.374^**^	−0.022	0.351^**^	0.409^**^	0.839^**^	1	
Sleep disorder	0.228^**^	0.307^**^	0.295^**^	0.403^**^	0.385^**^	0.004	0.343^**^	0.725^**^	0.922^**^	0.896^**^	1

**P < 0.01.

### 3.4 Mediating effect analysis of job burnout on the relationship between workplace violence and sleep disorders

The results demonstrated that the path coefficient (β) from workplace violence to job burnout was 1.658, indicating a significant predictive effect of workplace violence on job burnout (*p* < 0.01). The path coefficient from job burnout to sleep disorders was 0.132, indicating a significant predictive effect of job burnout on sleep disorders (*p* < 0.01). The path coefficient from workplace violence to sleep disorders was 0.250, indicating a significant direct predictive effect of workplace violence on sleep disorders (*p* < 0.01). The results of the mediating effect test showed that the total mediating effect of job burnout accounted for 46.7% of the total effect of workplace violence on sleep disorders among emergency department nurses. This indicates that job burnout plays a significant mediating role in the relationship between workplace violence and sleep disorders. See Tables 4–6 for the mediating effect results.

**Table 4:** Regression analysis of the mediation model of job burnout.

Variables	Model 1		Model 2		Model 3	
	*β(β’)*	*t*	*β(β’)*	*t*	*β(β’)*	*t*
Workplace violence	0.469(0.295)	12.126**	1.658(0.529)	24.477**	0.250(0.158)	5.645**
Job burnout					0.132(0.160)	9.300**
*R*	0.295		0.529		0.369	
*R^2^*	0.087		0.280		0.136	
*F*	147.038**		599.135**		120.847**	

**P < 0.01. Model 1: Workplace violence predict sleep disorder; Model 2: Workplace violence predict job burnout; Model 3: Workplace violence and job burnout jointly predicting sleep disorder.

**Table 5:** Tests for total, direct, and indirect effects of the variables.

Variable	Effect	*B*	*SE*	95%*CI*		%
				LLCI	ULCI	
Total effect	0.469	0.039	12.126	0.393	0.545	
Direct effect	0.250	0.044	5.645	0.163	0.337	53.30%
Indirect effect	0.219	0.025	8.760	0.170	0.268	46.70%

**Table 6:** Bootstrap path effect test.

Pathway	*β*	Boot SE	95%*CI*	
			LLCI	ULCI
Workplace violence → Job burnout	1.658	0.072	1.515	1.797
Job burnout → Sleep disorder	0.132	0.014	0.104	0.160
Workplace violence → Sleep disorder	0.250	0.046	0.162	0.341

## 4 Discussion

The results of this study revealed that the detection rate of sleep disorders among emergency department nurses was 913/1540 (59.2%). The prevalence of sleep disorders among emergency department nurses surpasses that reported for healthcare workers in general (2, 30) and aligns closely with the prevalence noted in nursing staff overall (31). Discrepancies in prevalence rates may stem from variations in study duration, participant demographics, and assessment methodologies employed in the studies. Nonetheless, it is evident that emergency department nurses encounter compromised sleep quality (4), which can detrimentally impact work performance (32) and elevate susceptibility to both psychological and physiological health issues (3). The issue of sleep disorders among nurses demands sufficient attention, and there is an urgent need for targeted interventions to improve the sleep quality of emergency department nurses.

The study reveals a direct link between workplace violence and sleep disorders in emergency department nurses. Around 77% of emergency department personnel encounter workplace violence (33), with younger and less experienced individuals being particularly vulnerable (34). A research conducted in Egypt also discovered that workplace violence is linked to decreased sleep quality among healthcare workers (35). Workplace violence not only hampers nurses’ job performance by disrupting their capabilities (36) but also triggers early neuropsychological symptoms like headaches and sleep disorders (37). This investigation confirms the positive correlation between workplace violence and sleep disorders, a connection observed not only in nurses but also in various occupational sectors, especially within healthcare professionals. Subsequent studies should delve into the efficacy of interventions designed to alleviate the association between workplace violence and sleep disorders. Such investigations would offer a solid basis, both theoretically and practically, to enhance occupational health for emergency nurses. Establish a three-level intervention framework from violence prevention to sleep repair, including work environment improvement, training programs for solving nurse–patient conflicts, stress management workshops, and sleep health education.

Job burnout mediates the relationship between workplace violence and sleep disorders in emergency department nurses, consistent with Havaei et al.’s (38) findings where burnout mediated the link between workplace violence and health outcomes like anxiety and sleep disorders in medical-surgical nurses. Previous research suggests that job burnout is a consequence of workplace violence, while sleep disorders result from burnout. Exposure to workplace violence activates the hypothalamic–pituitary–adrenal (HPA) axis, leading to increased physiological stress levels that can trigger sleep disorders (39). In patients experiencing burnout, methylation levels of the BMAL1 gene in the suprachiasmatic nucleus (SCN) show a negative correlation with sleep efficiency (40). Substance P, a neuropeptide identified by Nicoletti et al. (41), modulates anxiety, stress, and regulates sleep duration. This indicates that stress perception, regulation, and sleep patterns are influenced by common underlying factors, supporting burnout’s mediating role in the relationship between workplace violence and sleep disorders. Interestingly, among correctional officers, sleep problems mediate the connection between workplace violence and burnout (42), suggesting a potential bidirectional influence between burnout and sleep problems, which may vary across populations with different occupational characteristics.

## 5 Conclusion

Both workplace violence and job burnout directly associated with sleep disorders in emergency nurses. Furthermore, workplace violence can influence emergency nurses’ sleep disorders through the mediating role of job burnout. Nursing managers should prioritize maintaining a harmonious and safe workplace environment in the emergency department and implementing interventions targeting job burnout, thereby improving sleep disorders among emergency nurses. In addition, the effect of relevant interventions can be carried out. For example, the effectiveness of mindfulness-based interventions in high-stress nursing environments and targeting specific populations, such as emergency department nurses.

## 6 Limitation

Firstly, as a single cross-sectional survey focused primarily on emergency nurses, the conclusions of this study require further validation among nurses in other departments. Secondly, this study was filled out by the emergency nurse self through electronic questionnaires, and there may be self-report bias and selection bias. Finally, some confounding factors, such as previous mental health status, drug use or family stress were not assessed. Future research should employ longitudinal designs to examine the impact trajectories of workplace violence on job burnout and sleep disorders among emergency nurses.

## Data Availability

The raw data supporting the conclusions of this article will be made available by the authors, without undue reservation.
